# Peer driven or driven peers? A rapid review of peer involvement of people who use drugs in HIV and harm reduction services in low- and middle-income countries

**DOI:** 10.1186/s12954-021-00461-z

**Published:** 2021-02-03

**Authors:** Judy Chang, Shaun Shelly, Machteld Busz, Claudia Stoicescu, Arif Rachman Iryawan, Dinara Madybaeva, Yuri de Boer, Andy Guise

**Affiliations:** 1 International Network of People Who Use Drugs, INPUD Secretariat, Unit 2C09, South Bank Technopark, 90 London Road, London, SE1 6LN UK; 2 South African Network of People Who Use Drugs; University of Pretoria, Cape Town, South Africa; 3 Mainline, Amsterdam, The Netherlands; 4 School of Social Work, Columbia University, New York, USA; 5 HIV/AIDS Research Centre, Atma Jaya University, Jakarta, Indonesia; 6 Rumah Cemara, Bandung, Indonesia; 7 AFEW Kyrgyzstan, Bishkek, Kyrgyzstan; 8 AFEW International, Amsterdam, The Netherlands; 9 King’s College London, London, UK

**Keywords:** Rapid review, Community, Community involvement, People who use drugs, HIV, Harm reduction, Peer, Peer involvement

## Abstract

**Introduction:**

Peer involvement of people who use drugs within HIV and harm reduction services is widely promoted yet under-utilised. Alongside political and financial barriers is a limited understanding of the roles, impacts, contexts and mechanisms for peer involvement, particularly in low- and middle-income settings. We conducted a rapid review of available literature on this topic.

**Methods:**

Within a community-academic partnership, we used a rapid review approach, framed by realist theory. We used a network search strategy, focused on core journals and reference lists of related reviews. Twenty-nine studies were included. We developed thematic summaries framed by a realist approach of exploring interventions, their mechanisms, outcomes and how they are shaped by contexts.

**Results:**

Reported outcomes of peer involvement included reduced HIV incidence and prevalence; increased service access, acceptability and quality; changed risk behaviours; and reduced stigma and discrimination. Mechanisms via which these roles work were trust, personal commitment and empathy, using community knowledge and experience, as well as ‘bridge’ and ‘role model’ processes. Contexts of criminalisation, under-resourced health systems, and stigma and discrimination were found to shape these roles, their mechanisms and outcomes. Though contexts and mechanisms are little explored within the literature, we identified a common theme across contexts, mechanisms and outcomes. Peer outreach interventions work through trust, community knowledge and expertise, and ‘bridge’ mechanisms (M) to counter criminalisation and constraining clinic and service delivery environments (C), contributing towards changed drug-using behaviours, increased access, acceptability and quality of harm reduction services and decreased stigma and discrimination (O).

**Conclusion:**

Peer involvement in HIV and harm reduction services in low- and middle-income settings is linked to positive health outcomes, shaped by contexts of criminalisation, stigma, and resource scarcity. However, peer involvement is under-theorised, particularly on how contexts shape mechanisms and ultimately outcomes. Efforts to study peer involvement need to develop theory and methods to evaluate the complex mechanisms and contexts that have influence. Finally, there is a need to expand the range of peer roles, to embrace the capacities and expertise of people who use drugs.

## Introduction

Peer involvement in HIV and harm reduction programmes is widely promoted as essential to effective responses to the health, social, and political challenges faced by people who use drugs [1, 2]. Despite the recognition of meaningful community involvement as a principle, in practice there is a consistent lack of funding and political support [3]. Alongside ideological and financial barriers, peer involvement within harm reduction services has received less research attention, especially in the Global South. There is, therefore, a need to review and critically assess the evidence-base for peer involvement in order to better support ongoing research, policy and advocacy debates.

From the very beginnings of harm reduction, people who use drugs have been central to its development and delivery [4]. Studies, mainly focused on high-income settings, support the positive impact of different forms of peer involvement on service reach, accessibility and quality, as well as on the lives of peers themselves [4–10]. The varied engagement of peers follows a long history of peer-organising in regions including North America, Western Europe, Oceania and East and South-East Asia, either working behind the scenes or at the forefront of needle distribution services, harm reduction education, peer support and community-based research initiatives [11–20]. However, the available evidence centers on justifying the broader approach or outcomes, rather than on the specific operations and contexts for peer-involved programming. Past reviews note the limited understanding of the processes and mechanisms for this work, particularly in low- and middle-income countries (LMICs) [4, 21, 22]. Addressing this gap, particularly within low and middle-income settings, could enhance contemporary debates on peer involvement within global health and development.

Currently, there is a disjuncture between high-level support for peer involvement and the limited role of peers in practice. Through their endorsement of the 2016 Political Declaration on HIV/AIDS, UN member states committed to allocating 30% of funding for HIV programmes towards community-led responses by 2030 [23]. Aligned with this goal, communities of key populations, that is gay and bisexual men, people who inject drugs, sex workers and transgender people published *Implementation Tools* promoted community-led initiatives [2, 24–26] in collaboration with UN agencies, which have been recognised as global normative guidance by multi-lateral funding agencies [27]. Nonetheless, progress by policymakers and programme managers toward meaningfully involving people who use drugs in HIV and harm reduction programming remains limited, as evidenced by ongoing debates within the UNAIDS Programme Coordinating Board on how to operationalise the 30% target on funding community-led responses in the 2016 Political Declaration on HIV/AIDS [28]. Based on our personal participation in these policy debates, we consider that an enhanced understanding of the evidence for operations and impacts of peer involvement could be a core step in the successful operationalisation of the principle of peer involvement.

In summary, there is a need to review the evidence on peer involvement in HIV and harm reduction services in low- and middle-income countries. The lack of a comprehensive understanding of the impacts and processes for peer involvement constrains the development and operationalisation of best practice and prevents external agencies from identifying appropriate and effective means of support. It is precisely this lack of understanding, particularly on impact in low and middle-income countries, that we seek to address through our rapid review.

## Methods

The aims and questions of this review were determined within a community-academic research collaboration following principles of community-based participatory research (CBPR) [29]. The study was led by content experts from global and national community-based organisations, coalescing the combined expertise of lived experience, advocacy and programming with academic social science research.

Reflecting time and resource constraints, as well as the policy orientation of this paper, we used a rapid review approach [30]. This approach balances comprehensiveness with pragmatism and assuages the tensions between the time-sensitive demands and information needs of policy makers and the resource requirements of conventional systematic reviews [31]. We drew on realist theoretical approaches to rapid reviews [32, 33]. Pawson and Tilley (1997) argue that evaluations aiming to be useful to decision-makers must endeavour to answer ‘what works in which circumstances and for whom’, rather than merely ‘does it work’ [34], by exploring the situational contexts, mechanisms and outcomes that may enable or constrain the implementation of a given intervention. Identifying these factors and their interactions can generate transferable ‘program theories’ underpinned by Context-Mechanism-Outcome (CMO) configurations for planned interventions [34].

Rapid reviews still maintain a commitment to being systematic and rigorous. Guided by the PRISMA guidelines, we followed the core steps of being driven by a research question, using clear and reproducible methods and reporting all areas of the review [30]. The review had five iterative stages: 1) Development, 2) Search and collation of literature, 3) Screening, 4) Appraisal, 5) Synthesis and analysis and 6) Impact and dissemination. A reporting checklist based on rapid review guidelines [30] is included in Appendix 1.

### Development

The scope of this review was discussed and agreed over several meetings of the community-academic team. Questions, study aims, inclusion and exclusionary criteria were agreed upon by the lead authors. Research questions were guided by realist logic, as well as concerns brought about by our collective experiences. The overarching aim of the review to explore peer involvement in harm reduction services in low and middle-income countries was linked to the following sub-questions: What roles or forms do community involvement take within harm reduction services? What are the potential impacts and outcomes on service access and quality? What are the mechanisms for these roles? How does context shape these roles and outcomes?

### Search

Our literature search was driven by a network approach [35]. We built on related reviews, as well as our prior knowledge of the literature. We used the related reviews [4, 21, 36] as index papers and identified potential papers from their bibliographies. We added papers that we were familiar with and hand-searched leading journals in the field, *Addiction, Drug and Alcohol Review, Harm Reduction Journal, International Journal of Drug Policy*, and *Social Science and Medicine* using the terms ‘community’, ‘harm reduction’ and ‘peer’. From our searches conducted between April and June 2018, we identified 48 papers for consideration.

### Selection

**Fig. 1:**
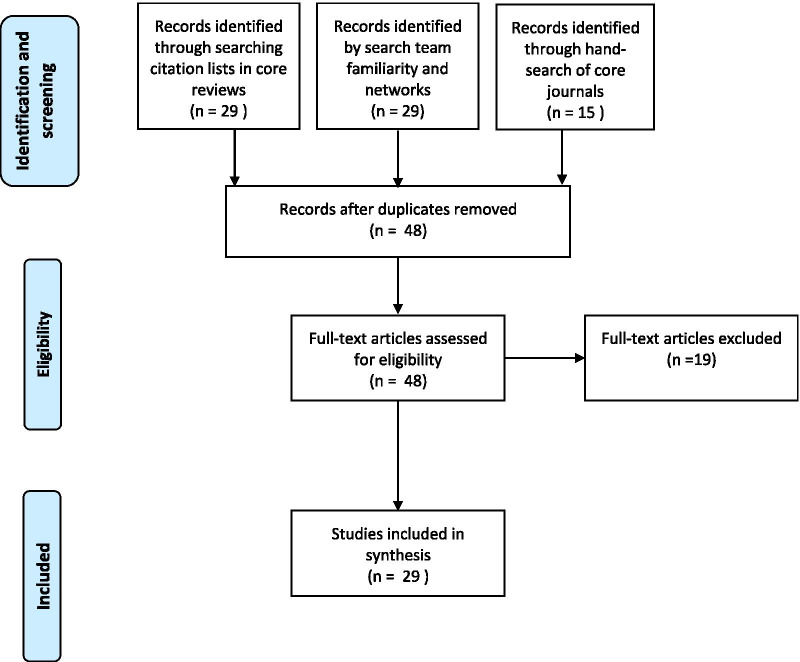
From: Moher D, Liberati A, Tetzlaff J, Altman DG, The PRISMA Group (2009). Preferred Reporting Items for Systematic Reviews and MetaAnalyses: The PRISMA Statement. PLoS Med 6(7): e1000097. 10.1371/journal.pmed1000097

We used a double review process with the 48 papers divided amongst JC, SS and AG. All read and agreed on paper inclusion. We included qualitative and quantitative studies published in English that described the direct or indirect involvement of peers in harm reduction services in countries defined by the World Bank as low- or middle-income countries and excluded editorials and commentaries. We used a broad definition of harm reduction that encompasses the UNODC/WHO comprehensive package of services as well as other health and social services that aim to reduce drug-related harms [37]. Based on the above criteria, twenty-nine papers were retained covering a date range from 1998 to 2018 (Fig. 1).

### Appraisal

We did not perform a quality assessment using formal quality assessment tools, reflecting both ongoing debate on their utility [38] and the aims of our review to understand the overall nature of the evidence base and foster ongoing theory and policy debate. Instead, we assessed the included papers for appropriateness of language in terms of terminology judged problematic for potentially stigmatising attitudes and attentiveness to people-first terms i.e. people who use drugs.

### Analysis

We used a narrative approach whereby we sought to develop thematic summaries of the literature around a realist framework [39]. After a period of extensive discussion and open coding of the literature, we developed summaries of each paper’s core findings as they related to the realist framework and then drew out overlapping themes. We used an adapted version of Marshall et al.’s 5 part typology of peer involvement in harm reduction services to structure our analysis. We adapted the roles of 1) harm reduction education, 2) direct harm reduction and health services, 3) peer support, counselling and referrals, 4) research assistance, and 5) advisory committees. Research assistance, not directly linked to our review questions and addressed in prior reviews [3] was therefore excluded. Given the focus of the research, we replaced 4) and 5) with management and advocacy. We identified recurring patterns across the contexts, mechanisms and outcomes for each role and then also sought to identify recurring themes linking particular roles to impacts, mechanisms and contexts (i.e. CMO configurations) [38]. The development of themes was led by one author and validated by a second.

### Impact and dissemination

This rapid review aims to bridge the evidence gap on impacts of peer-involvement in HIV and harm reduction programmes in low- and middle-income countries. Making use of the role of the authors as advocacy practitioners, the results of the study will be promoted and advanced with a view to translate the evidence into actionable commitments. Emerging plans and initial themes from the review were shared at meetings, workshops and conferences attended by the authors, including the 2018 International AIDS Society (IAS) conference in Amsterdam and the 2019 International Network on Hepatitis in Substance Users (INHSU) conference. We intend to further use the review to shape policy debates and discussions at the global and national levels – particularly on how community involvement is defined, understood and operationalised by UN agencies and member states.

## Findings

### Overview of included papers

We included twenty-nine papers, summarised in Table 1. Two studies were set in sub-Saharan Africa, in Kenya [40] and Senegal respectively [41]. Seventeen papers were undertaken in the Asia–Pacific region; three from China [42–44], six studies from Vietnam [45–50], four in Thailand [51–54] and India respectively [55–58]. Five studies were set in Eastern Europe and Central Asia; four in Ukraine and one in Russia [59–63]. Five studies were multi-country comparative research projects set across China and Vietnam, as well as across different regions [64–68]. In terms of methods applied, eleven studies were quantitative—five were randomised controlled trials (RCT) and six were mixed method studies. Six qualitative studies utilised a range of methods, including interviews and ethnographic observation.

**Table 1:** Included papers

	Reference	Design	Country
1	Ayon, S., et al. (2017). "Barriers and facilitators of access to HIV, harm reduction and sexual and reproductive health services by women who inject drugs: role of community-based outreach and drop-in centers." AIDS Care: 1–8	Qualitative study	Kenya
2	Bartlett, N., et al. (2011). "A qualitative evaluation of a peer-implemented overdose response pilot project in Gejiu, China." International Journal of Drug Policy 22(4): 301–305	Qualitative study	China
3	Booth, R. E., et al. (2016). "HIV incidence among people who inject drugs (PWIDs) in Ukraine: results from a clustered randomised trial." Lancet HIV 3(10): e482-489	Cluster randomized trial	Ukraine
4	Booth, R. E., et al. (2009). "Use of a Peer Leader Intervention Model to Reduce Needle-Related Risk Behaviors among Drug Injectors in Ukraine." Journal of Drug Issues 39(3): 607–625	Survey with baseline and follow-up linked to intervention implementation	Ukraine, Crimea
5	Booth, R. E., et al. (2011). "Individual and Network Interventions With Injection Drug Users in 5 Ukraine Cities." American journal of public health 101(2): 336–343	Survey with baseline and follow-up linked to intervention implementation	Ukraine
6	Broadhead, R. S., et al. (2009). "Peer-Driven Interventions in Vietnam and China to Prevent HIV: A Pilot Study Targeting Injection Drug Users." Journal of Drug Issues 39(4): 829–850	Survey with baseline and follow-up linked to intervention implementation	Vietnam and China
7	Des Jarlais, D. C., et al. (2007). "Reducing HIV infection among new injecting drug users in the China–Vietnam Cross Border Project." AIDS 21: S109-S114	Serial cross sectional survey	cross broder between China and Vietnam
8	Dhand, A. (2006). "The roles performed by peer educators during outreach among heroin addicts in India: ethnographic insights." Social Science & Medicine 63(10): 2674–2685	Ethnography	India
9	Friedman, S. R., et al. (2007). "Harm reduction theory: Users’ culture, micro-social indigenous harm reduction, and the self-organization and outside-organizing of users’ groups." International Journal of Drug Policy 18(2): 107–117	Secondary analysis of qualitative and quantitative data and studies	New York City, Rotterdam, Buenos Aires, and sites in Central Asia
10	Go, V. F., et al. (2013). "Effects of an HIV peer prevention intervention on sexual and injecting risk behaviors among injecting drug users and their risk partners in Thai Nguyen, Vietnam: A randomized controlled trial." Social Science & Medicine 96: 154–164	Randomised Controlled Trial	Vietnam
11	Hammett, T. M., et al. (2012). "Controlling HIV Epidemics among Injection Drug Users: Eight Years of Cross-Border HIV Prevention Interventions in Vietnam and China." PLoS ONE 7(8): e43141	Serial cross-sectional surveys, interviews and HIV tests	Vietnam and China
12	Hayes-Larson, E., et al. (2013). "Drug users in Hanoi, Vietnam: factors associated with membership in community-based drug user groups." Harm Reduction Journal 10(1): 33	Survey	Vietnam
13	Hoffman, I., et al. (2013). "A Peer-Educator Network HIV Prevention Intervention Among Injection Drug Users: Results of a Randomized Controlled Trial in St. Petersburg, Russia." AIDS and Behavior 17(7): 2510–2520	Randomised Controlled Trial	Russia
14	Jain, B., et al. (2014). "Effect of peer-led outreach activities on injecting risk behavior among male drug users in Haryana, India." Harm Reduction Journal 11(1): 3	Programme data	India
15	Kerr, T., et al. (2010). "Expanding the reach of harm reduction in Thailand: Experiences with a drug user-run drop-in centre." International Journal of Drug Policy 21(3): 255–258	Cross-sectional survey	Thailand
16	Kumar, M. S., et al. (1998). "Community-based outreach HIV intervention for street-recruited drug users in Madras, India." Public Health Reports 113(Suppl 1): 58–66	Randomised Controlled Trial	India
17	Latkin, C. A., et al. (2009). "The efficacy of a network intervention to reduce HIV risk behaviors among drug users and risk partners in Chiang Mai, Thailand and Philadelphia, USA." Social Science & Medicine 68(4): 740–748	Randomised Controlled Trial	USA and Thailand
18	Le, L. T., et al. (2015). "Coalition building by drug user and sex worker community-based organizations in Vietnam can lead to improved interactions with government agencies: a qualitative study." Harm Reduction Journal 12(1): 38	Qualitative study	Vietnam
19	Liu, B., et al. (2007). "An evaluation of needle exchange programmes in China." AIDS 21: S123-S128	Cross sectional survey	China
20	Moorthi, G. (2014). "Models, experts and mutants: Exploring the relationships between peer educators and injecting drug user clients, in Delhi's harm reduction programs." Qualitative Social Work 13(1): 69–84	Ethnography	India
21	Ngo, A. D., et al. (2009). "Qualitative evaluation of a peer-based needle syringe programme in Vietnam." International Journal of Drug Policy 20(2): 179–182	Qualitative study	Vietnam
22	Sherman, S. G., et al. (2009). "Evaluation of a peer network intervention trial among young methamphetamine users in Chiang Mai, Thailand." Social Science & Medicine 68(1): 69–79	Randomised Controlled Trial	Thailand
23	Smyrnov, P., et al. (2012). "Rejuvenating harm reduction projects for injection drug users: Ukraine's nationwide introduction of peer-driven interventions." International Journal of Drug Policy 23(2): 141–147	Programme data	Ukraine
24	Stengel, C. M., et al. (2018). " “They accept me, because I was one of them”: formative qualitative research supporting the feasibility of peer-led outreach for people who use drugs in Dakar, Senegal." Harm Reduction Journal 15(1): 9	Qualitative study	Senegal
25	Ti, L., et al. (2012). "HIV testing and willingness to get HIV testing at a peer-run drop-in centre for people who inject drugs in Bangkok, Thailand." BMC Public Health 12	Cross-sectional survey	Thailand
26	Ti, L., et al. (2013). "Low rates of hepatitis C testing among people who inject drugs in Thailand: implications for peer-based interventions." J Public Health (Oxf) 35(4): 578–584	Cross-sectional survey	Thailand
27	Van Khoat, D., et al. (2003). "Peer Education for HIV Prevention in the Socialist Republic of Vietnam: A National Assessment." Journal of Community Health 28(1): 1–17	Review of annual reports, combined with qualitative study	Vietnam
28	Walsh, N., et al. (2009). "The development of peer educator‐based harm reduction programmes in northern Vietnam." Drug and Alcohol Review 27(2): 200–203	Rapid assessment linked to programme reflections	Vietnam
29	Wang, K., et al. (2014). "Do community-based strategies reduce HIV risk among people who inject drugs in China? A quasi-experimental study in Yunnan and Guangxi provinces." Harm Reduction Journal 11(1): 15	Survey	China

### Quality review

A majority of the papers were limited with respect to community-endorsed language. Only three studies used person-first language in writing up their studies [40, 42, 44]; the majority of studies using medicalised or psychiatric language, most commonly using acronyms ‘IDU’ or reverting to ‘addict’ language, carrying connotations of the irrational, compulsive and disordered subject [69–72]. A caveat is that many of the studies were published more than a decade ago, prior to a growing chorus on the need to use more neutral, inclusive and de-pathologising language [70].

## Results

We first summarise the roles peers took aross the studies, then the mechanisms, outcomes and contexts for these roles; then we discuss specific context-mechanism-outcome (CMO) configurations.

### Roles of peers in HIV and harm reduction services

Harm reduction education, direct services, and peer support, counselling and referrals were the roles most commonly discussed, often in combination.

The role of harm reduction education for peers was documented across all twenty-nine studies included. These activities included HIV prevention education and overdose prevention awareness/education.

Peer distribution of services and commodities was mentioned in nineteen studies and consistently linked to the delivery of harm reduction education [40–47, 50, 51, 53–58, 65–67]. Concerning direct service provision, peers operated hotlines and distributed condoms, needles and syringes.

Marshall’s third category, peer support, counselling and referrals was described in sixteen studies [40–42, 44, 45, 47, 49–51, 54–58, 62, 64]; and included peer support groups, counselling and peer navigation, and providing social and economic support. This was usually done in tandem with harm reduction education and direct service provision.

Only seven studies reported on the role of peers in the management of service organisations, including the organic organisation of responses and formal organisation through peer-led programmes and services [44, 46, 49, 51, 53, 54]. Of these seven, three studies reported on peer-led advocacy [45, 49, 51].

### Mechanisms

Trust between peer workers and clients was a crucial mechanism. Four studies featured trust and related features such as empathy and commitment [40–42, 58]. Peers drew on trust to effectively communicate information and engage with clients. In a peer-run overdose prevention programme in China, Bartlett [44] stated that staff were were "better prepared", "more experienced", had a "non-discriminatory and responsible attitude" and were "more likely to ensure confidentiality" (p. 303).

Six studies articulated community knowledge and experience as the mechanism via which peer roles productively function [40–42, 49, 55, 67]. Peer workers had similar life events and embodied social and psychological experiences which helped them better understand the needs of clients and help navigate contexts of stigma, criminalisation and programmatic barriers.

Four studies articulated the role that peers play as that of a ‘bridging’ role, where peer workers act as a bridge between clients and programmes [40, 41, 49, 58]. Because of their knowledge and experiences of common practices across social contexts, peer workers are able to link together the different ‘worlds’ of clients and services and undertake a process of mediation, managing differences and tensions.

Three studies discuss the ‘role model’ mechanism in describing how peer roles function [41, 55, 58]. In this role, peer workers draw on their personal experience to provide mentoring advice and model behaviours for peers to follow. However, being a ‘role model’ may also have some troubling, but little examined, implications for the differential power dynamics enacted between peer workers and clients. This ‘vertical positioning’ can inculcate resentment and isolation [48, 49], individually responsibilising peers for their own health, ‘risk reduction’ and personal ‘recovery’ [56].

### Outcomes

Six studies, of which two were randomised control trials, documented changes in health status linked to peer involvent. Four studies reported a reduction in HIV incidence and prevalence as a result of involving peers [42, 52, 60, 63, 65, 66]. Booth et al.’s (2016) found that a peer leader network intervention, as compared to counselling and testing only, was associated with reduced HIV incidence in Ukraine; a similar study in Russia found a less clear effect [63]. An evaluation of an eight-year cross-border project aimed at reducing HIV risk behaviours, incidence and prevalence by Hammett et al. [66] concluded that combining peer education and widespread needle and syringe distribution in harm reduction programming led to significant decreases in HIV prevalence across three cities in China and Vietnam (Lang Son 46% to 23%, Ning Ming 17% to 11% and Ha Giang 51% to 18%). Two other studies reported high rates of successful resuscitation with naloxone after overdose in a peer-run service [42] and reductions in STI incidence following a peer-network intervention, although with similar effect to a best-practice life skills intervention [52].

Fourteen studies reported how peer approaches impacted on HIV risk behaviours across seven countries [43–46, 48, 52, 56, 57, 59, 61, 64, 66–68]. The resulting risk reduction practices were safer injecting practices, including a reduction of injecting with others, usage of common containers and sharing equipment.

Thirteen studies found that peer approaches, with peer educators providing health information and materials, peer support and counselling and direct provision of commodities, increased access to, acceptability and quality of HIV and health prevention care and treatment services for people who use drugs [40, 42–45, 47, 49–51, 53, 54, 62, 67]. For instance, Smyrnov et al. [62] show that peer-driven interventions were powerful in recruiting people to harm reduction services, including women and young people. Several studies documented greater acceptability of services delivered through peer-based education and distribution models, leading to increases in HIV and HCV testing and distribution of HIV prevention and health tools [40, 44, 47, 51, 53, 54, 67].

Three studies reported changes in stigma and discrimination [40, 46, 50]. Three articles documented a lessening in stigma and discrimination and improvements in community attitudes away from reified notions of drug use as a ‘social evil’ due to peer involvement in service delivery [40, 46, 50].

### Context

Eight of the studies found that criminalisation, including fear of arrest, detention and harassment was linked to peer involvement [42, 43, 46, 49–51, 64, 67]. People who use drugs are not only driven away from services, but peer workers are also targeted by police, resulting in high attrition rates of programme staff and limits capacity to support services [42, 46, 51, 64]. In Vietnam, Le et al. [49] reported that beneficial collaboration was hampered because peers could mistrust peer workers who were seen to be collaborating with the government due to their status as staff of government-run harm reduction programmes.

Clinic and service delivery environments are mentioned in ten of the review papers [40–43, 46, 47, 49, 55, 58, 67]. The studies went on to describe the challenges, including a lack of trust in health services, the costs of healthcare and lack of funding and political will for harm reduction.

A common challenge identified within programmes involving peers was inequitable pay structures between peers and ‘professionals’. Such inequities generate tensions, which subsequently affect peer empowerment, retention of peers, and ultimately, programme sustainability [41, 43, 46, 47, 55, 58].

Five studies pointed towards stigma and discrimination as particular challenges within clinic and service delivery environments, as well as in community settings, where peers experience negative and judgemental attitudes that undermine their engagement with services [40, 42, 46, 50, 67].

### Context-mechanism-outcome configurations

Relationships between peer roles and then context, mechanisms and outcomes were rarely explored within studies. The majority of the literature excluded direct analysis of either contexts or mechanisms, a point we return to in the discussion section.

Exceptions are found in three studies. In Ayon et al. [40], peer educators provide peer support and counselling, deliver services and make referrals (I) which function through trust, bridging and peer’s community knowledge (M) that offset difficult clinic and service delivery environments, including experiences of stigma and discrimination (C), resulting in reduced stigma and discrimination and increased access and quality of health services (O)’. As one peer expressed, ‘When you get to the hospital they connect you to health care workers. The outreach worker will tell them your problem, then you get treatment’ (^40^, p. 483). In Bartlett et al.’s [42] article evaluating a peer overdose prevention programme, the trust, commitment and empathy, community knowledge and experience, and ‘bridge’ mechanisms (M) enabled peer outreach workers (I) to overcome contextual challenges. Due to criminalisation and clinic and service delivery environments, people accessing services avoided hospitals due to fears of police attention and incarceration, stigma and discrimination and high costs (C). Since peers are ‘better prepared, more experienced and faster to respond, had a non-discriminatory and responsible attitude and were more likely to ensure confidentiality than the paramedics’ (^42^, p. 303), this led to outcomes of increased access and quality of services (O). Similarly Le et al. [49] found drug user-led networks (I) overcame the challenge of mistrust between peers and law enforcement and peer mistrust of the health system (C) through mechanisms of community knowledge and experience and bridging (M), ultimately leading to outcomes (O) of decreased stigma and increased access, acceptability and quality of services.

Based on our findings, the following CMO theme emerges. Peer educators and outreach workers, by acting as educators, service delivery and referral agents (I) mitigate the effects of contextual barriers of criminalisation and fear of arrest, stigma and discrimination, as well as clinic and service delivery barriers such as fees, distance and lack of confidentiality (C), through mechanisms of trust, empathy and commitment; community knowledge and expertise and their role as ‘bridges’ between health clinics and peers (M). This enhances and contributes towards outcomes of reduced stigma and discrimination as well as heightened access and quality of harm reduction services (O).

## Discussion

Through our narrative synthesis of literature exploring peer involvement in harm reduction services in low and middle-countries, we identified a range of roles, mechanisms, outcomes and contexts. Peer involvement principally involves roles in harm reduction education, peer counselling and service referrals, often in combination; less commonly studied are peer roles in management and advocacy. Although outcomes in the literature were sometimes inconclusive, not studied or found to have no positive influences, and contexts and mechanisms in some studies were insufficiently discussed, meaning that a deeper understanding of how outcomes are reached and the social realities in which interventions are embedded were not articulated or anlaysed, CMO configurations specifying how peer interventions work and their value within harm reduction could still be identified.

A core challenge we identify based on this review is the frequently limited role for peers in harm reduction services. Peer roles are often marginal and instrumentalised, whereby peers achieve objectives set only by others, negating and under-recognising the capacities and knowledge peers can bring. Some studies explore more nuanced ways in which peers can be engaged in a meaningful and influential manner, such as through peer empowerment, collectivisation and advocacy, revealing the potential for truly ‘peer-driven’ services [42, 45, 49, 51, 53, 67]. More commonly however, the situation is one of ‘driven peers’ responding to limited roles, opportunities, support and resources and beset by challenges of low or no pay and limited support. Whilst roles in peer education, counselling and commodity distribution represent a core way in which people who use drugs can contribute to the objectives of harm reduction, such roles more commonly reflect how peers respond to priorities and goals set by others. This theme is further compounded by the small subset of literature exploring the roles of peer involvement in service management and advocacy, both of which afford peers agency and autonomy [44, 46, 49, 51, 53, 54]. Across the included studies we also note how the peer role can be linked to ‘moral-auditing’: ‘ex-users’ are set up in opposition to ‘current users’, given preferential hire, expected to model ‘recovery’ behaviours and rewarded with more prestigious positions within the programmes. This responsibilisation of peers towards ‘recovery’ threatens not only to amplify divisions between past and current users of drugs, but can ultimately limit the scope, scale and effectiveness of harm reduction interventions through limiting the potential for trust and empathy. In these ways, peers are commonly driven by values, preferences and political systems that constrain and limit their agency and empowerment both as individuals and as a collective, placing restraints on mechanisms that facilitate impacts and outcomes of peer involvement.

The overall characteristics of the evidence-base are reflective of the particular norms circulating amongst policy and health care professionals and the unequal power dynamics between these actors and people who use drugs. Barriers and challenges to more enhanced peer roles include disparate payment structures, working conditions, and limited or lack of support, all of which act to generate tensions, undermine peer empowerment, and negatively impact on the retention of peers and the delivery of services [41, 43, 55, 58]. Allowing for the delivery and evaluation of more expansive and complex forms of peer involvement rests on ensuring institutional support and resources for these programmes. This should comprise agreed standards as set out by peer-led networks, including appropriate pay, respectful and flexible working conditions and action on stigma and discrimination [73]. Peer-led networks, with people who use drugs in the driver’s seat as board members, directors and managers have successfully provided accessible, flexible and high-quality HIV and harm reduction services across multiple settings [2].

The emphasis on ‘driven peers’ across the literature is also related to dominant norms and methodologies for research and evaluation. The prominence placed on evaluating harm reduction education and similar interventions can be linked to discourses of evidence-based policy that bias familiar and demarcated interventions [74, 75]. Sociological researchers have critiqued the reification of the evidence-based policy making endeavour for its assumption of neutrality and objectivity, masking the ‘discourse privilege’ of certain kinds of ‘rational voices’ and practices to the exclusion of others, such as people who use drugs [76, 77]. Researchers, and research funders, also need to reflect on their methodologies and theoretical frameworks for evaluation, to allow better engagement with a range of forms of peer involvement, such as community leadership and management, which might be harder to evaluate given challenges around ethics and complexity of the interaction between CMO and their links to multiple outcomes [78–82].

Extensive global study has demonstrated how contextual factors produce health and social risks for people who use drugs, and challenge the availability of, and access to, appropriate harm reduction services [83–87]. This review validates this trend by demonstrating how contextual factors including criminalisation, stigma and service delivery environments both shape and limit peer involvement. These factors should however be attended to much more in future research design by, for example, seeking aligned action on structural enablers of criminalisation, such as legal and policy change, and addressing endemic stigma and discrimination through public campaigns. Whilst the included studies indicated how programmes could adapt to challenging contexts, we also found relatively little research engagement with context. Many studies omitted the role of criminalisation, stigma and poverty or other contextual factors within their analytical frameworks. Therefore, alongside changes in how services are designed and supported, researchers and research institutions should adapt their methods and theoretical frameworks to enhance understanding of the influences of context, and how these can be mitigated.

A further characteristic of the literature we surveyed was limited theorising of peer involvement in how harm reduction services operate. Whilst some studies referenced underlying theory for the effects described [48, 58, 63], most studies were limited in theorising how particular roles led to various mechanisms, outcomes, and under the influence of particular contextual conditions. This limitation undermines the potential for effective learning of lessons across different settings and limits the scope of evaluation within evidence-based oriented policy debates. There are multiple challenges to rigorous, theory-based evaluation of peer involvement in harm reduction services, including time, resources and institutional buy-in; these challenges are not easily addressed. However, supporting the ongoing development of theory as it relates to this field is essential for enhancing policy and research debates.

## Recommendations

Our review has three principal implications. First, policy debates need to engage more with the breadth of evidence for peer involvement in low- and middle-income countries, and through this explore how peer involvement can be more fully and meaningfully operationalised. Services and future research should engage with suggested strategies to support peer involvement: address criminalisation and stigma, enable networks of people who use drugs and champion their relationship-building with policy makers and researchers, foster organisational cultures and resources that respectfully utilise peer knowledge and skills; and address the multiple barriers to the participation of people who use drugs in services through action on the social determinants of health.

Second, the range of roles for peers in harm reduction services remains limited, under-utilising the strengths and expertise of the community and under-serving the scope and reach of existing programming. Broader exploration of peer roles including, but not limited to, advisory committees, programme management, and advocacy would mutually benefit communities and service providers.

Third, research and the overarching effort to evaluate peer involvement needs to adapt theoretically and methodologically to the specific operations of peer involvement. Researchers, policymakers, and people who use drugs must engage in debates seeking to both further theory and methods, but also bring more nuance on what is considered appropriate evidence to support policy change. New ways of engagement that shift power, make space for new forms of knowledge-making and praxis should be sought, allowing all parties to maximise potential.

### Strengths and limitations

This review has several strengths and limitations. The rapid review approach, by design, and the need to reconcile diverse approaches and limited timelines, involves pragmatic choices on search and analysis and places some limits on rigour. Our review nonetheless provides important insight to a currently marginalised debate within policy and research. The strengths of the review include a community-led effort to explore debates on the underlying mechanisms of peer involvement and how these link to outcomes. Our community-academic partnership sought to ensure that the design and interpretation of the review responded to the needs, concerns and expertise of people who use drugs across international settings.

## Conclusions

The review underscores that the involvement of people who use drugs in programming, particularly within low- and middle-income settings, remains an area needing priority and support. With the advent of COVID-19, the role of the social and structural determinants of health, including community-level action, has become more evident than ever, creating new opportunities for advancing the debate, politics and practices around meaningful community involvement [88, 90]. Our review makes evident that peer involvement within harm reduction programmes can have positive impacts on health outcomes, including disease incidence and prevalence, through a range of mechanisms shaped by overarching contextual factors. Progressing and consolidating evidence-making on the roles, mechanisms and contexts and their interaction provides further justification to advance the agenda on meaningful peer involvement. Existing global commitments, such as the 30% target of funding community-led responses within the 2016 Political Declaration on HIV/AIDS, need to be operationalised by member states and strongly advocated for by civil society. Operalisation of international commitments requires, first and foremost, the expansion and diversification of roles for people who use drugs within policy, programmes and research processes. Peers need to be acknowledged not only as representatives of the communities they are part of, but as peers of the policymakers, researchers, clinicians and professional staff they work alongside and advise daily.

## Data Availability

All data generated or analysed during this study—in the form of cited studies—are included in this published article.
